# A Case of Multisystem Inflammatory Syndrome in an Immunosuppressed Adult

**DOI:** 10.7759/cureus.25635

**Published:** 2022-06-03

**Authors:** Abrahim N Razzak, Abhishek Janardan, Ipsit Shah, Pinky Jha, Yasir Abdelgadir

**Affiliations:** 1 School of Medicine, Medical College of Wisconsin, Milwaukee, USA; 2 Internal Medicine, Medical College of Wisconsin, Milwaukee, USA

**Keywords:** multisystem inflammatory syndrome in adults, kidney transplantation, covid-19, sars-cov-2 (severe acute respiratory syndrome coronavirus 2), corticosteroid treatment, immunosuppression and covid-19, coronavirus disease 2019 (covid-19)

## Abstract

Multisystem inflammatory syndrome in an adult (MIS-A) is a rare immunological complication that gained prominence after the coronavirus disease 2019 pandemic. Patients with MIS-A often clinically present with non-specific generalized symptoms, such as fever, myalgia, or fatigue, making the diagnosis difficult. In this article, we present an unusual case of MIS-A in a 50-year-old male that raises the question of whether the immune system’s dysregulation will demonstrate differing criteria of signs and symptoms for a patient on sustained immunosuppression as opposed to the non-immunosuppressed population.

## Introduction

Multisystem inflammatory syndrome in adults (MIS-A) is a rare and underdiagnosed complication that follows coronavirus disease 2019 (COVID-19) after approximately four weeks [1]. This condition is difficult to diagnose due to the myriad of non-specific generalized symptoms. Most notably, MIS-A usually presents as cardiovascular shock and gastrointestinal symptoms without significant respiratory illness [2]. Affected patients are commonly ethnic minorities, middle-aged or young adults, and previously healthy individuals [3]. MIS is more common in children (MIS-C) and unevenly distributed among races and ethnicities with greater incidences in Hispanic, Asian, and Black children [4]. The typical clinical presentation of MIS-A is fever and signs of inflammation [5]. While the identification of MIS-A is difficult, elevated inflammatory markers including C-reactive protein (CRP), ferritin, D-dimer, and interleukin (IL)-6 serve as valuable indicators for clinicians [6]. At present, definitive treatment for MIS-A remains unclear. Current guidelines suggest a treatment protocol using pulsed steroids, intravenous immunoglobulin, advanced heart failure devices, and vigorous supportive care in an intensive care unit [7,8]. Here, we present a case of MIS-A in an immunosuppressed patient who was treated with corticosteroids with positive outcomes and discharged without end-stage organ damage.

## Case presentation

A 50-year-old Caucasian male with a medical history of kidney transplantation surgery, ankylosing spondylitis, type 2 diabetes mellitus, reactive leukocytosis, acute kidney injury, hypertension, depression, and chronic pain presented to the emergency department (ED) in April 2022 with intermittent high grade (103°F) fever of unknown origin, myalgia, chills, and fatigue. The patient’s immunosuppressants after the transplantation surgery included 5 mg of prednisone daily, 1.5 mg of tacrolimus daily, and 1,000 mg of mycophenolate mofetil twice a day.

In February 2022, the patient contracted a COVID-19 upper respiratory tract infection but his symptoms resolved. However, the patient re-tested positive with COVID-19 in late March 2022, the second time with a lower viral load, during which he developed fevers, chills, and myalgias without respiratory symptoms. While the patient’s symptoms improved briefly for a week after the infection, his high-grade fever and fatigue returned a couple of weeks post his March infection prompting an outpatient infectious disease specialist visit. The patient was worked up by the infectious disease specialist a few days before the ED encounter. At the time, computerized tomography (CT) of the chest, blood cultures, urine *Streptococcus pneumonia* antigen, *Legionella*, and urine *Histoplasmosis* antigen were unremarkable. Inflammatory markers were elevated with CRP at 19 mg/L and ferritin at 511.0 ng/mL. At the time of ED presentation, the patient denied productive cough, nasal congestion, runny nose, sore throat, headaches, photophobia, neck pain, joint pain, skin rash, oral/genital ulcers, or diarrhea. He also denied significant respiratory or abdominal symptoms.

The patient was admitted to the floor from the ED and extensively worked up for infectious, autoimmune, or malignant causes, which all came back negative. These tests included *Streptococcus*/*Legionella* urine antigen test, *Cryptococcal/Histoplasma* antigen test, COVID-19 nucleic acid amplification test (NAAT), human immunodeficiency virus and Epstein-Barr virus test, chest X-ray, CT of the abdomen/pelvis, extended respiratory panel, renal ultrasound, transthoracic echocardiogram, blood draw labs, urine analysis, and urine culture. Of note, the patient had leukocytosis, neutrophilia, lower hematocrit, high blood glucose levels, and a low effective glomerular filtration rate during the visit (Table 1). The patient also had a moderate elevation of inflammatory markers with CRP, ferritin, and erythrocyte sedimentation rate (ESR) (Table 1). After consultation with the transplant infectious diseases team, the patient was suspected to have MIS-A and was not prescribed antibiotics.

**Table 1:** Abnormal lab values during floor presentation.

Lab examination (units)	Patient results	Reference range
White blood cell count (1/µL)	13.4 × 10^3^	3.9 × 10^3^–11.2 × 10^3^
Neutrophil (%)	86	43–74
Hematocrit (%)	33	40–51
Blood glucose (mg/dL)	378	70–180
Glomerular filtration rate (mL/minute)	50	≥60
C-reactive protein (mg/dL)	11.57	<0.50
Ferritin (ng/mL)	403.0	30.0–400.0
Erythrocyte sedimentation rate (mm/hour)	63	0–32

During three days of in-patient workup and supportive treatment, corticosteroids (IV methylprednisolone 80 mg daily during admission) were used as a diagnostic and therapeutic protocol, leading to an overall improvement of the patient’s condition. Without evidence of end-stage organ damage, the steroids improved the patient’s symptoms to a stable condition for recovery and discharge. Given the patient’s extensive medical history, the rheumatology team was consulted. Steroids were instructed to be tapered off post-discharge with 40 mg prednisone orally twice a day for a week, followed by 20 mg prednisone orally twice a day for the week and 20 mg prednisone orally daily the week after. The patient was instructed to take Bactrim prophylaxis and refrain from his etanercept during the prednisone taper. Given the findings of lower blood pressure, the patient was also recommended to hold off on blood pressure medications, such as amlodipine and doxazosin, and resume the medications when systolic blood pressure was above 140 mmHg. The patient was instructed to continue the rest of his medications including the insulin dosing chart regimen, tacrolimus 1.5 mg daily, and mycophenolate mofetil 1,000 mg twice a day alongside coordination with a primary care provider. While renal function improved, the patient was recommended to follow up with outpatient transplant nephrology for magnetic resonance imaging of the right kidney alongside recovering acute kidney injury. The patient was reached out four days post-discharge and discussed that his condition continues to be stable and that he was following the necessary treatment protocols.

## Discussion

The constellation of symptoms prompting the diagnosis of MIS is a relatively recent discovery. First identified in April 2020 in children, the following months noted similar findings among adults. Although the true incidence rates of MIS-A are unknown, it is considered to be rare. Temporally, MIS-A commonly occurs within a span of four to six weeks after a diagnosed COVID-19 episode; however, a specified timeline of onset has not been conclusively established [1,3].

The Centers for Disease Control and Prevention defines the following guidelines for the diagnosis of MIS-A: a patient aged ≥21 years hospitalized for ≥24 hours meeting the ensuing clinical measures. Primary clinical criteria required for the diagnosis of MIS-A include the involvement of fever prior to the hospitalization or within the first three days of hospitalization alongside either a severe cardiac illness or rash and non-purulent conjunctivitis. Secondary clinical criteria involve the presence of either new-onset neurological symptoms, shock or hypotension not attributable to medical therapy, abdominal pain, vomiting, or diarrhea and thrombocythemia. Laboratory evidence supporting the diagnosis of MIS-A needs to include elevated measurements of at least two of the following markers of inflammation: D-dimers, procalcitonin, ferritin, IL-6, ESR, and CRP [9].

The predominant ethnic and demographic groups that presented with MIS-A infections were non-Hispanic blacks or Hispanic males between the ages of 19 and 34 [1]. MIS-A presents clinically with a plethora of disseminated symptoms. Patel et al. summarized some of the most common findings to be fever, hypotension, cardiac dysfunction, dyspnea, diarrhea, and elevated inflammatory markers [1].

Our patient’s clinical findings do not fit within the established criteria for diagnosis of MIS-A. Our patient suffered from fevers, myalgia, chills, and fatigue during admission and endorsed two infections with COVID-19 shortly prior to hospital admission. Our patient also displayed elevated levels of CRP and ferritin. However, the classic primary and secondary clinical symptoms involving the cardiac, dermatological, neurological, and gastrointestinal systems were not seen.

Our case represents one of the rare recorded instances of MIS-A possibly arising within the setting of a patient with a history of a kidney transplant. There has been one noted example of inflammatory syndrome following COVID-19 in a kidney transplant recipient according to our literature search. Our patient also displayed atypical findings. This case involved the patient presenting with a peaking fever shortly following admission. The patient’s respiratory condition was exacerbated due to COVID-19 pneumonia, necessitating treatment with oxygen therapy, azithromycin, and ceftriaxone. The treatment regimen displayed minimal improvement in clinical function, resulting in the transition to tocilizumab and dexamethasone followed by high-dose steroid therapy. This newer treatment protocol proved to be more efficacious, yielding tremendous improvement in the patient’s clinical functioning. The patient presented a few weeks later with de novo atrial fibrillation and bilateral knee pain. Anti-SARS-CoV-2 antibody test was positive. Blood tests indicated elevated inflammatory markers: CRP was measured at 150 mg/L, ferritin at 3,749 μg/L, and IL-6 at 629 pg/mL. Screening for infections, hematological malignancies, and autoimmune markers yielded negative results. Treatment with intravenous immunoglobulin immediately improved inflammatory symptoms [10].

There are many similarities that exist between our case and the case described above. Both instances of MIS-A involved kidney transplant patients receiving immunosuppression therapy with prednisone and mycophenolic acid. Both patients tested negative for infectious agents, autoimmune markers, and hematological malignancies. Both patients did not display extensive primary and secondary clinical symptoms outlined for the diagnosis of MIS-A. The clinical outcomes of both patients improved with corticosteroids. MIS-A is thought to arise due to a delayed, dysregulated immune response [1]. An interesting area of study would be to analyze how this dysregulation manifests among patients with a history of sustained immunosuppression. With the immune system not functioning at the level expected of the stereotypical demographic of MIS-A, a healthy young adult male, the clinical presentation can be thought to be vastly different, as noted in the instances of MIS-A among patients with a history of kidney transplants.

## Conclusions

MIS-A is a rare immunological complication that gained prominence following the COVID-19 pandemic. The current diagnostic criteria for MIS-A include fever, myalgia, fatigue, rash, neurological symptoms, severe cardiac illness, shock, and elevated inflammatory markers. While this case did not completely fit the MIS-A diagnostic criteria, the case raised the question of whether a medical history of immunosuppression affects the immune response’s dysregulated manifestation in the case of MIS-A as opposed to the non-immunosuppressed population. Given the similarities in symptom presentation and corticosteroid treatment between MIS-A cases for patients with kidney transplants, the question of different symptom presentations based on the immunosuppressed population should be taken into future consideration regarding the critera for MIS-A diagnosis.
